# The Role of the Host Cytoskeleton in the Formation and Dynamics of Rotavirus Viroplasms

**DOI:** 10.3390/v16050668

**Published:** 2024-04-25

**Authors:** Janine Vetter, Melissa Lee, Catherine Eichwald

**Affiliations:** Institute of Virology, University of Zurich, 8057 Zurich, Switzerland; janine.vetter@uzh.ch (J.V.); melissa.lee2@uzh.ch (M.L.)

**Keywords:** rotavirus, viroplasm, cytoskeleton, microtubule, actin, molecular motors, lipid droplets, NSP5, NSP2, VP2, VP4

## Abstract

Rotavirus (RV) replicates within viroplasms, membraneless electron-dense globular cytosolic inclusions with liquid–liquid phase properties. In these structures occur the virus transcription, replication, and packaging of the virus genome in newly assembled double-layered particles. The viroplasms are composed of virus proteins (NSP2, NSP5, NSP4, VP1, VP2, VP3, and VP6), single- and double-stranded virus RNAs, and host components such as microtubules, perilipin-1, and chaperonins. The formation, coalescence, maintenance, and perinuclear localization of viroplasms rely on their association with the cytoskeleton. A stabilized microtubule network involving microtubules and kinesin Eg5 and dynein molecular motors is associated with NSP5, NSP2, and VP2, facilitating dynamic processes such as viroplasm coalescence and perinuclear localization. Key post-translation modifications, particularly phosphorylation events of RV proteins NSP5 and NSP2, play pivotal roles in orchestrating these interactions. Actin filaments also contribute, triggering the formation of the viroplasms through the association of soluble cytosolic VP4 with actin and the molecular motor myosin. This review explores the evolving understanding of RV replication, emphasizing the host requirements essential for viroplasm formation and highlighting their dynamic interplay within the host cell.

## 1. Rotavirus

Rotavirus (RV) was initially observed in 1963 via electron microscopy of feces samples of young monkeys and mice presenting diarrhea [1]. In humans, the virus was first described in 1973 in the duodenal mucosa of infants with acute nonbacterial gastroenteritis [2]. Fifty years later, RV infections are the leading cause of severe gastroenteritis and dehydration in infants and young animals [3]. In 2008, before worldwide RV vaccine programs, RV gastroenteritis led to 435′000 deaths worldwide, mainly in developing countries, and high-cost hospitalization in developed countries [4]. The introduction of vaccine programs reduced the disease burden by 85% in developed countries [5]; however, developing countries show a much more modest reduction in disease burden [6].

RV is a nonenveloped virus belonging to the order of *Reovirales* within the family *Sedoreoviridae*, where it forms the genus *Rotavirus* [7,8]. The genus *Rotavirus* currently entails nine different RV species, A-D and F-J, distinguished by serological criteria, host range, and sequence analysis [9]. The strains designated as RV species E were nonrecoverable from long-term storage, and no sequence information is available to support its existence, being consequently removed from the RV species list by the ICTV in 2019 [10]. Moreover, recent reports indicate the identification and sequencing of RV species K and L [11,12]. However, the ICTV has not yet approved them.

The mature RV virion is a nonenveloped, icosahedral (T = 13), triple-layered particle (TLP) [13] of about 100 nm in diameter [14]. The virion encapsidates one copy of each of the eleven double-stranded RNA (dsRNA) genome segments. Each genome segment encodes for one protein, six structural proteins (VP1, VP2, VP3, VP4, VP6, and VP7), which are incorporated into the mature virion, and five (or six) nonstructural proteins (NSP1, NSP2, NSP3, NSP4, NSP5, and, in certain strains, also NSP6) [15].

The spike protein, VP4, is cleaved in the intestine tract by a trypsin-like protease in two main products, VP8* (28kDa, amino acids 1-247) and VP5* (60 kDa, amino acids 248-776), that remain noncovalently associated with the infectious particle allowing the initiation of the RV entry [16,17]. In this context, VP8* initiates RV cell entry by attaching to various cellular glycans [18], among them terminal sialic acids [19,20] and histo-blood group antigens (HBGAs) [21]. After cell binding, RV favors entry to the cell via clathrin-mediated endocytosis, although some strains also use clathrin-independent pathways [22,23]. 

A common step in RV entry is the localization of the virus particles into early endosomes, where they are exposed to environmental changes, such as an acidic pH, low calcium concentrations, or other lysosomal components. Those factors seem to be involved in the entrance of the virus into the cytosol [24], but they appear to be strain-dependent, as some strains seem to profit from maturing endosomes, while others use late endosomes [25]. It is thought that VP5* is involved in forming pore-like structures in the endosomes, eventually allowing endosomal escape [26,27].

In this process, the outer layer of the virion is detached, and the double-layered particles (DLPs) are released in the cytosol. These DLPs become transcriptionally active [28], releasing into the cytosol capped, nonpolyadenylated (+)ssRNAs [29] for direct translation of the virus proteins required to (i) block the innate immune response of the host (NSP1 and VP3) and (ii) build viroplasms (NSP5, NSP2, and VP2) [30,31,32,33]. Moreover, increased levels of virus mRNA transcripts inhibit the translation of the host polyadenylated mRNAs [34].

Specific RV proteins accumulate within specialized cytosolic inclusions called viroplasms, where newly synthesized RV genome segments are packaged in newly formed cores, followed by the addition of a middle coat layer to form DLPs. Subsequently, DLPs exit the viroplasms via a not fully understood pathway. The current proposed mechanism involves the association of VP6 with NSP4 embedded in the membrane of the endoplasmic reticulum (ER) [35,36]. Simultaneously, the spike protein VP4 localizes between the viroplasm and the ER, associating with NSP4 [37]. These associations with NSP4 bring VP6 and VP4 in proximity, leading to the formation of a transiently enveloped DLP (eDLP) in the lumen of the ER [38]. In fact, eDLP reconstructions appear as DLPs with 60 trimeric VP4 spikes, which connect the particles to the transient envelope where VP7 and NSP4 are not discernible in images [38].

The assembly is completed in a poorly understood process by incorporating the outer-layer protein VP7, which is present in the ER [37,38]. The fully formed TLPs are then released either via cell lysis [39] or in an actin-dependent process from the cell surface [40,41,42].

## 2. Viroplasms

### 2.1. Spatial and Temporal Organization

**Figure 1:**
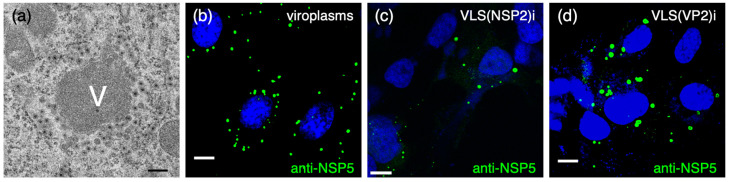
Comparison of viroplasms and VLSs. (**a**) Electron micrograph of viroplasm (V) at 6 hpi. The electron dense viroplasm structure is surrounded by the endoplasmic reticulum membrane filled with TLPs at diverse stages of maturation. Scale bar is 200 nm. Immunofluorescence of images of viroplasm (**b**), VLS (NSP2)i (**c**), and VLS (VP2)i (**d**) immunostained with anti-NSP5 (green). Nuclei are stained with DAPI (blue). Scale bar is 10 µm for **b**–**d**.

Viroplasms are membraneless globular electron-dense cytosolic inclusions (Figure 1a). These structures are responsible for virus genome replication and the generation of new rotavirus virions. So far, only RVA has been shown experimentally to induce viroplasm formation; their formation in other species remains to be demonstrated. The viroplasms comprise NSP5, NSP4, NSP2, VP6, VP1, VP2, VP3, virus single- and double-stranded RNA, and host components such as tubulin, perilipin, the host proteasome, and cellular chaperonins [28,43,44,45,46,47,48]. Finally, viroplasms are adjacent to the ER, enriched in VP4 and VP7 [43,49]. 

Viroplasms are detected as early as 2 h postinfection (hpi), and their size steadily increases [50,51]. Interestingly, the number of viroplasms per cell decreases at 6 hpi, indicating coalescence between the structures and their dynamic nature [50,52]. Indeed, it has recently been shown that viroplasms are liquid-like inclusions [53]. Furthermore, during the infection, viroplasms move toward the perinuclear region of the cell in a process that is dependent on the microtubule (MT) cytoskeleton [52].

Remarkably, the expression of NSP5 in the presence of NSP2 or VP2 induces the formation of viroplasm-like structures (VLS) [54]. VLSs closely resemble the morphology of RV viroplasms (Figure 1b–d). Due to their simplicity, VLSs are a valuable model for studying viroplasms within a host using an in vivo approach, as they share characteristics such as coalescence and perinuclear condensation [50,54].

### 2.2. Replication Steps within Viroplasms

The exact mechanism of genome packaging and assembly of virions within the viroplasms is unclear. The current model assumes that pregenomic (+)ssRNAs are organized sequence-specifically through the assistance of NSP2 [55] in the viroplasms and packaged in the assembling core while simultaneously being replicated into dsRNA [56]. The filled cores move towards the periphery of the viroplasms, which are rich in VP6 for converting the cores into DLPs. These DLPs would produce (i) more (+)ssRNA [57] or (ii) migrate to the ER to become mature TLPs [58].

### 2.3. NSP5

Inhibition of NSP5 expression via RNA interference completely abolishes viroplasm formation and synthesis of genomic dsRNA as well as progeny virus, revealing the essential role of NSP5 in viroplasm formation [59]. While the complete structure of NSP5 remains unknown, it has been shown to form dimers and oligomers through its C-terminal region [60,61]. In this context, NSP5 has been shown to be an intrinsically disordered protein [60], which is consistent with a high propensity to phase separation of the viroplasms [53]. NSP5 is a hyperphosphorylated protein in infected cells, achieved by phosphorylation by cellular kinases, such as casein kinase (CK1alpha), and regulated via autoregulation and interaction with NSP2 and VP2 [62,63,64,65,66,67]. The phosphorylation cascade is critically dependent on the presence of a serine at position 67 [62]. A recombinant RV (rRV) harboring a point mutation in NSP5 in serine 67 to alanine (S67A) shows aberrant viroplasms. This observation suggests that hyperphosphorylation of NSP5 is crucial for viroplasm morphology [68]. Additionally, the study highlights the significance of the NSP5 tail region in the phosphorylation cascade and viroplasm formation [63,68]. Despite the initial notion that autokinase activity could be described, no kinase activity could be attributed to NSP5, which, in addition to being the primary driver of viroplasm formation, displays ATPase activity [61,63,69].

### 2.4. NSP2

Another critical protein in viroplasm formation is NSP2. Similarly to NSP5, inhibition of NSP2 expression also leads to impairment of viroplasm formation [70]. NSP2 self-assembles into donut-shaped octamers, as denoted with crystallographic and cryogenic electron microscopy analyses for species A, B, and C [71,72,73,74]. These multimers can interact with the RNA-dependent RNA polymerase VP1 and viral RNA [75,76]. Furthermore, it has been demonstrated that NSP2 is an RNA chaperone, capable of binding to RNA transcripts and consequently controlling their interaction and unfolding [77]. NSP2 has been linked to several enzymatic activities, among them a nucleoside diphosphate kinase-like activity [78], RNA-helix-destabilizing activities [78], and nucleoside triphosphatase (NTPase) activity [71]. NSP2 plays a direct role in viroplasm coalescence events [50]. NSP2 is found to be dispersed in the cytosol (dNSP2), and its phosphorylated version is exclusively found in the viroplasms (vNSP2) [79,80]. The phosphorylation of NSP2 occurs uniquely in S313 by CK1 alpha. Notably, the phosphorylation of NSP2 has been implicated in viroplasm formation, as evidenced by the delayed formation of viroplasms observed in a rRV harboring an NSP2 S313D phosphomimetic mutant [81]. Additionally, studies using a mutant NSP2 harboring a lysine-to-glutamic acid change in the C-terminal region revealed the importance of a flexible tail in viroplasm biogenesis and coalescence properties [82].

### 2.5. VP2

An often-overlooked protein in the context of viroplasms is VP2. Silencing of VP2 expression in infected cells reduces the number of viroplasms per cell [83]. VP2, primarily studied as the main structural core protein, is also an inducer of VLS formation when co-expressed with NSP5 [44]. It has been shown that VLS formation is critically dependent on the presence of the three amino acids, L124, V865, and I878, with residues highly conserved in VP2 of RV species A-H [84]. Previous studies have demonstrated that NSP2 [64] and VP2 [44,84] trigger the hyperphosphorylation cascade of NSP5. Additionally, VP2 has been implicated in modifying viroplasm perinuclear localization [52].

## 3. Host-Cell Cytoskeleton

### 3.1. Microtubules

MTs are a significant component of the cytoskeletal network in eukaryotic cells, forming a dynamic network of polymeric filaments distributed throughout the cytoplasm. MTs play pivotal roles in numerous cellular processes, such as cell division, intracellular transport, motility, and organelle positioning. MTs are hollowed-out tubes formed from α-tubulin and β-tubulin (αβ-tubulin) heterodimers that are polarized and typically oriented toward the cell periphery [85]. The polarity, a crucial requirement for MT function, results from the head-to-tail polymerization of tubulin dimers with α-tubulin at the minus end and β-tubulin at the plus end [86]. Notably, individual filaments can reach up to 5000 µm persistence length in vitro, much longer than actin filaments, which can only reach persistence lengths of 15–20 µm [87].

An exciting feature of tubulins is their ability to undergo various reversible post-translational modifications (PTMs), such as acetylation, phosphorylation, polyglycylation, polyglutamylation, (de)tyrosination, and palmitoylation [88,89]. Most PTMs occur in the carboxy-terminal tails of tubulin, with the notable exception of acetylation [89]. Acetylation mainly occurs after the assembly of MTs and is associated with stabilizing the MT structure [89]. In addition, acetylation can improve the binding and transport of molecular motors, such as kinesin-1 or dynein [90,91]. Another way to regulate MT functions is with nonmotor MT-associated proteins (MAPs), classified as MT-stabilizers, destabilizers, or plus-end tracking proteins [92,93]. MAPs also play a major role in MT bundling, a process that further regulates the stability of MT filaments [94,95].

The MT cytoskeleton is exploited by numerous viruses throughout almost all stages of the viral life cycle [96], including internalization [97], viral factory formation [98], assembly [99], and virus release [100].

### 3.2. MT-Dependent Molecular Motors

Two main classes of molecular motors specialize in transport along the MT network, corresponding to kinesin and dynein motors. Kinesin motors move towards the MT plus-end in what is known as anterograde transport. The diverse cargoes can either associate directly with the heavy chain or bind to specific regions in the C-terminus of the light chain [101]. In contrast, the molecular motor dynein moves towards the MT minus end, performing retrograde transport [102]. The cargo can bind to dynein in numerous ways, allowing for a wide range of client proteins [103,104]. Viruses, as cargoes, exploit cytoplasmic dynein to facilitate their transport within the cell [101].

### 3.3. Actin

Actin is the most abundant protein in many eukaryotic cells. Accordingly, several viruses subvert the actin cytoskeleton to spread and move over long distances [105].

Actin is expressed as a globular monomer known as G-actin [106]. When it polymerizes, it forms F-actin, filamentous structures that can form spontaneously in physiological conditions. Actin fibers play a fundamental role in many cellular processes, including motility, morphogenesis, cytokinesis, or endocytosis [107]. Actin-bundling proteins can crosslink actin filaments into actin bundles, which are the main components of the actin network [108]. When smaller filaments are organized into microvilli in the plasma membrane protrusions and tightly packed into arrays, the filaments are referred to as brush borders [109]. Within the cells, the force of actin is produced by myosin molecular motors that move along the long actin domains, referred to as stress fibers [107,110]. These stress fibers are often anchored to focal adhesions corresponding to complex structures responsible for crucial scaffolding interactions with actin [111].

### 3.4. Actin-Dependent Molecular Motors

Over forty classes of myosins are expressed in eukaryotes, divided into muscle and nonmuscle myosins [112]. Known as “conventional myosin,” nonmuscle myosin-2 (NM2) is present in almost every cell type, existing in three variants [113]. Therefore, it is not surprising that NM2 has been shown to play a role in the life cycle of numerous viruses [112].

### 3.5. Intermediate Filaments

The intermediate filaments (IFs) are the third component of the eukaryotic cytoskeletal network and are less studied than MTs and actin [114]. One reason is that the IFs are polymers of two, three, or more different proteins. These proteins include, among others, keratins, vimentin, lamins, and nestin, which form six subtypes of filaments [114]. Vimentin and nestin play a role in cell migration, but other proteins have diverse functions depending on the cell context [115]. Interestingly, IFs are formed in the cytoplasm and the nucleus [116]. So far, no motor proteins have been identified moving along IFs.

## 4. Viroplasm Interaction with the Host Cytoskeleton

Aside from the viral components, viroplasms interact with many cellular components, including lipid droplets, proteins, and host nucleic acids. In this context, viroplasms are found to recruit components of lipid droplets (LDs) during the replication cycle [117]. LDs are spherical organelles that play a significant role in lipid homeostasis and contain mostly perilipins [118]. Associations with LDs appear to be required to form viroplasms and infectious virus progeny by serving as a scaffold for viroplasm assembly and allowing the association between viroplasms and ER membranes [45,117,119]. 

However, viroplasms are also found to interact with many elements of the host-cell cytoskeleton. All three primary cytoskeletal components (actin, MTs, and IFs) are restructured during RV infection. The formation of viroplasms relies on several of these reorganizations [120,121,122,123,124,125].

**Figure 2:**
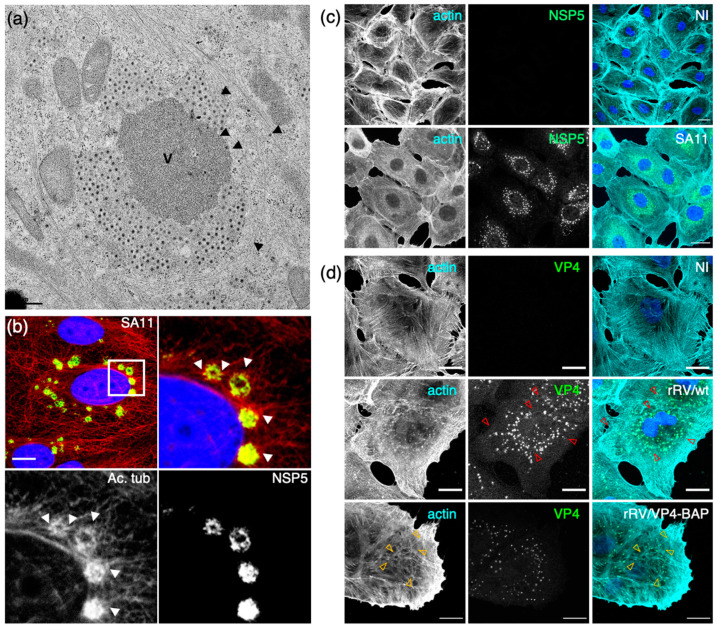
Association of viroplasms with microtubules and actin. (**a**) Electron microscopy of SA11-infected MA104 cells at 8 hpi, showing viroplasm. Black arrowheads indicate the MT bundles; viroplasms (V). Scale bar is 0.5 µm. (**b**) Immunofluorescence of SA11-infected MA104 cells at 6 hpi showing viroplasms (anti-NSP5, green), acetylated tubulin (mAb antiacetylated tubulin, red) and nucleus (DAPI, blue), upper left image. The white-boxed area shows an enlarged photomicrograph indicating the localization of the hyperacetylated MTs (white arrowheads) in the viroplasm region. Scale bar is 15 µm. From Eichwald et al., 2012 [52]. (**c**) Immunostaining of noninfected and SA11-infected MA104 cells. At 6 hpi, cells were fixed with methanol and immunostained to detect viroplasms (anti-NSP5, green) and actin cytoskeleton (antiactin, cyan). Nuclei were stained with DAPI (blue). The scale bar is 20 μm. Immunostaining of noninfected and rRV/wt- or rRV/VP4-BAP-infected MA104 cells. At 6 hpi, cells were fixed with methanol and immunostained for detection of (**d**) VP4 (anti-VP4, green) and actin cytoskeleton (antiactin, cyan). Nuclei were stained with DAPI (blue). The scale bar is 20 μm. Open yellow and red arrowheads point to stress fibers in the actin cytoskeleton and VP4 fibers, respectively. From Vetter et al., 2022 [142].

The reorganization of the MT cytoskeleton has been shown to directly influence the coalescence and localization of viroplasms [52], which seems a trait common among many viruses inducing the formation of membraneless replication compartments, such as birnaviruses, reoviruses, or African swine fever viruses [98,126,127,128,129]. In this sense, MT depolymerization drugs harm both perinuclear condensation and coalescence of the viroplasms. On the other hand, MT stabilizing drugs, such as taxol, showed no effect. In fact (Figure 2a,b), RV infection increases stabilized MTs, as denoted by the rise of acetylated tubulin in viroplasms [52]. Collectively, RV can subvert the cytoskeleton to assemble and maintain viroplasms.

Indeed, RV NSP2 and NSP5 have been implicated in directly interacting with tubulin in coimmunoprecipitation assays followed by Western blot or mass spectrometry. However, while the interaction of NSP2 with tubulin appears very stable, the interaction between NSP5 and tubulin is shown to be weak [52,80,130,131]. NSP5 has been pulled down with tubulin as a contaminant in RV-infected cells due to its ability to bind to NSP2 [50,60]. It seems that NSP5 and tubulin compete for binding to the same positively charged grooves on the NSP2 octamer [130]. Interestingly, despite significant MT reorganization induced by NSP2 transfection, the study does not observe considerable colocalization of NSP2 and tubulin in NSP2-transfected cells [130]. Furthermore, NSP2 exhibits a robust binding to nonacetylated tubulin compared to acetylated tubulin [80]. Still, acetylated tubulin seems to accumulate in mature viroplasms [52]. 

A newly identified variant of NSP2 displays varying interactions with NSP5 and acetylated tubulin, depending on the phosphorylation status of NSP2 [80]. These two NSP2 conformations have been distinguished using two different monoclonal antibodies targeting different regions of NSP2. One conformation corresponds to viroplasmic NSP2 (vNSP2), which localizes in viroplasms. The second conformation is a cytosolic dispersed pool of NSP2 (dNSP2), which is phosphorylated at its C-terminus, specifically in S313. Additionally, dNSP2 is weakly colocalizing with NSP5 and vNSP2 in viroplasms. Interestingly, dNSP2 resulted in the unphosphorylated precursor of vNSP2, where dNSP2 is phosphorylated by CK1 alpha to generate vNSP2. Once NSP2 is phosphorylated (vNSP2), it can bind to acetylated tubulin and NSP5. On the other hand, vNSP2 interacts with phosphorylated NSP5 and only weakly with tubulin [80]. This outcome suggests a mechanism of viroplasm formation and assembly coordinated by the phosphorylation of NSP5 and NSP2, with VP2 and tubulin acetylation. In this model, dNSP2, phosphorylated by CK1 alpha, and VP2 can bind nonphosphorylated NSP5, triggering NSP5 phosphorylation at Ser67, also by CK1 alpha, leading to the initial nucleation steps required for viroplasm formation. Both NSP2 and VP2 associate with unphosphorylated NSP5 [68,84]. These events concomitantly initiate the reorganization of the MT network to induce favorable conditions. Following this model, the destabilization of MTs during the early stages of infection hinders the coalescence of viroplasms [52]. Additionally, the globular morphology of the viroplasms seems dependent on the phosphorylation of NSP5, since unphosphorylated NSP5 leads to aberrant viroplasms [68]. However, VP2 also plays a role in the morphology of viroplasms, in which the inhibition of TRiC chaperonin leads to defective VLS composed of NSP5 and VP2 without affecting VLS composed of NSP5 and VP2, suggesting that the proper folding of VP2 is required for viroplasm structure [48]. Similarly, the expression of VP2 harboring L124 mutated to alanine leads to defective viroplasm formation [84]. 

Interestingly, the interaction with the MT network is not only based on NSP2–tubulin associations. In experiments using VLSs induced by coexpression of NSP5 with either NSP2 or VP2 and treated with an MT-destabilizing drug, it was shown that NSP2 confers the coalescence properties while VP2 mediates the perinuclear condensation properties. Additional research provides evidence that transfected NSP4 also binds and reorganizes the MT network [132,133,134]. Overall, the interaction of NSP5 and NSP2 with tubulin and their phosphorylation-dependent effects on viroplasm formation remain to be fully discovered.

Some studies point to the involvement of dynein-mediated transport in the coalescence of viroplasms [135]. NSP2 can interact with the dynein intermediate chain (DIC), mediating the ability of the viroplasm to coalesce. These findings resemble measles virus replication compartments, whose liquid–liquid phase-separated replication organelles depend on dynein-mediated transport to form large inclusion and viral replication [136]—suggesting a conserved reliance on dynein-mediated transport among diverse viruses to organize replication structures. In addition, it has been shown that viroplasms can no longer coalesce or move to the perinuclear region when the molecular motor Eg5 of the kinesin-5 family is inhibited [52]. So far, however, no direct interaction partner has been identified, as VLS properties seem to be independent of the Eg5 function, regardless of VLS induction by NSP2 or VP2 [52]. Moreover, RV infection halts the host cell cycle in the S/G2 phase [137], a stage that correlates with a stabilized MT network [138]. The RV-induced cell cycle arrest relies on the kinesin motor Eg5 and the actin and MT networks. This connection underscores the significance of a stabilized MT network for viroplasm formation, linking it with the cell cycle arrest and, consequently, RV replication [137].

The actin cytoskeleton plays an additional important role in viroplasm dynamics and formation. In this context, actin has mainly been found to interact with VP4, but NSP4 has likewise been shown to induce actin remodeling [139,140,141,142]. VP4 is predominantly known as a structural spike protein but is also expressed as a soluble protein in the cytosol [139]. The interaction of VP4 and actin is well known [42,139,141]. It has been found that VP4 can induce actin remodeling when expressed in the absence of other virus proteins [141]. Thus, VP4 has an actin-binding domain (ABD, amino acid region 713 to 773) at its C-terminus and a coiled-coil domain, allowing association to actin filaments. The VP4 ABD is buried in the assembled particle, pointing to the importance of soluble VP4 in the cytoplasm [139]. The use of a recombinant RV harboring a BAP tag in the VP8 region of VP4 (rRV/VP4-BAP) (Figure 2c,d) demonstrated that cytosolic VP4 plays a critical role, either directly or indirectly, in interacting with actin filaments to facilitate viroplasm formation [142]. Similarly, as observed for Negri bodies in rabies virus (RABV)-infected cells [143], the treatment with cytochalasin B, an inhibitor of actin filament dynamics, leads to a reduced number of viroplasms in RV-infected cells.

Additional studies proved that VP4 associates with MTs, potentially in an early step of virus release [144]. Studies have also shown that VP4 colocalizes with β-tubulin in both RV-infected and VP4-transfected cells, an interaction susceptible to disruption through MT depolymerization [144]. Moreover, it has been hypothesized that VP4 is transported to the plasma membrane via MT molecular motors [144]. It is plausible that the VP4 intracellular transport is differentially regulated, depending on the specific component of the cytoskeleton. It is well known that various viruses, such as flaviviruses [145] or influenza viruses [146], shift from actin-mediated transport to MT-associated transport at different steps of their life cycle. Transportation along the actin cytoskeleton may direct VP4 towards viroplasms to facilitate viroplasm formation. This process might involve the regulation of actin filaments and stress-fiber formation by VP4, which are necessary for initiating viroplasm assembly. In contrast, the MT network may transport VP4 away from viroplasms for incorporation in the plasma membrane in an alternative TLP assembly pathway [144]. The potential of VP4 to undergo differential transport opens new questions regarding the regulation of host-cell factors.

Only sparse research is available on the interplay of RV infection and the intermediate filaments. Infection with RV induces substantial restructuring of vimentin in adherent kidney cells, whereas such reorganization is not observed in differentiated human intestinal epithelial cells. Conversely, differentiated human intestinal epithelial cells display rearrangement of other cytoskeletal elements, a phenomenon not observed in undifferentiated human intestinal epithelial cells. [122,147]. Further research on the role of intermediate filaments is needed, as this is a relatively unexplored area. 

Despite significant progress in the research on the assembly and maintenance of viroplasms, there are still gaps in our understanding of the precise molecular mechanisms involved in their formation and organization, as well as the interplay between different cytoskeletal components and their regulatory mechanisms.

## 5. Interaction of Viral Factories with Host Components in Other dsRNA Viruses

Studies on other dsRNA viruses apart from RV, such as reoviruses or bluetongue virus, have revealed similar interactions between their viral factories and the host cell’s cytoskeleton. Notably, research on mammalian reovirus (MRV) viral factories indicates their reliance on the MT network for their perinuclear condensation, movement, and structural assembly [98], properties observable in RV viroplasm formation as well [52]. It was found that both filamentous and globular MRV viral factories need an intact MT network for proper function with dynein localizing in both viral factories [98]. Moreover, the MT network is essential for forming large globular perinuclear inclusions via MRV non-structural protein μNS, as nocodazole treatment, a tubulin depolymerizing agent, was shown to disperse the filamentous viral factories into smaller inclusions [148]. These results appear consistent with studies on RV showing inhibition of perinuclear condensation and coalescence upon treatment with nocodazole, suggesting similarities in the interaction of viroplasms with the host cytoskeleton in other dsRNA viruses [52].

Further, the interaction between reovirus core protein μ2 and MTs stabilized by bundling and hyperacetylation of α-tubulin determined the filamentous shape of reovirus inclusion bodies, highlighting the dependency of a stabilized MT network for the distribution of MRV viral factories in cells [127,149]. The direct association between MRV and spindle tubules observed in L2 cells could explain the aggregates of the virus in extensive perinuclear inclusions, although this association is not necessary for viral replication [150]. Furthermore, studies on both RV and MRV have shown that their infection disrupts and reorganizes vimentin filaments without affecting MTs or microfilament bundles [122,151]. Likewise, bluetongue virus associates with the cytoskeleton. Linear arrays of virus particles around viral inclusion bodies were found to be formed upon treatment with a chemical compound (colchicine), leading to aggregation of the vimentin filament network in the perinuclear region, suggesting an association of the viral inclusion bodies with the intermediate filaments [152].

## 6. Concluding Remarks

This review describes the crucial interactions between RV proteins and the cellular cytoskeleton. It has become clear that phosphorylation, particularly the sequence of phosphorylation events, and other PTMs play a critical role in regulating the interaction between RV proteins and the cytoskeleton, particularly between NSP5, NSP2, and tubulin. Additionally, the new role of VP4 in regulating viroplasm formation, through its interaction with actin filaments as previously described, underscores the multifunctionality of RV proteins. This highlights the significance of host-cell factors on the dynamics of viroplasms and virus replication.

In conclusion, this review underlines promising research areas and aims to enrich the ongoing discussion surrounding viroplasm assembly and maintenance. Addressing these unresolved questions and conducting further studies in these areas will deepen our comprehension of the complex interplay between RV and the host-cell cytoskeleton, potentially leading to the development of novel therapeutic strategies for combating RV infections.

## Data Availability

Not applicable.
